# Recombinant immunotoxins development for HER2-based targeted cancer therapies

**DOI:** 10.1186/s12935-021-02182-6

**Published:** 2021-09-06

**Authors:** Reza Mahmoudi, Hassan Dianat-Moghadam, Mansour Poorebrahim, Samaneh Siapoush, Vahdat Poortahmasebi, Reza Salahlou, Mohammad Rahmati

**Affiliations:** 1 Department of Medical Biotechnology, Faculty of Advanced Medical Sciences, Tabriz University of Medical Sciences, Tabriz, Iran; 2 Targeted Tumor Vaccines Group, Clinical Cooperation Unit Applied Tumor Immunity, German Cancer Research Center (DKFZ), Heidelberg, Germany; 3 Department of Bacteriology and Virology, Faculty of Medicine, Tabriz University of Medical Sciences, Tabriz, Iran; 4 Department of Clinical Biochemistry, Faculty of Medicine, Tabriz University of Medical Sciences, Tabriz, Iran

**Keywords:** Monoclonal antibody, Recombinant immunotoxin, Cytolytic fusion protein, Immunoapoptin, ImmunoRNase

## Abstract

Understanding the molecular mechanisms of cancer biology introduces targeted therapy as a complementary method along with other conventional therapies. Recombinant immunotoxins are tumor specific antibodies that their recognizing fragment is utilized for delivering modified toxins into tumor cells. These molecules have been considered as a targeted strategy in the treatment of human cancers. HER2 tumor biomarker is a transmembrane tyrosine kinase receptor that can be used for targeted therapies in the forms of anti-HER2 monoclonal antibodies, antibody–drug conjugates and immunotoxins. There have been many studies on HER2-based immunotoxins in recent years, however, little progress has been made in the clinical field which demanded more improvements. Here, we summarized the HER2 signaling and it’s targeting using immunotherapeutic agents in human cancers. Then, we specifically reviewed anti-HER2 immunotoxins, and their strengths and drawbacks to highlight their promising clinical impact.

## Background

Cancer hallmarks and their relevant molecular targets are the basis for development of novel treatment strategies [1]. Sustained proliferative signaling is one of the most fundamental hallmarks which upregulates the growth-promoting signals in cancer cells. Most of these signals are mediated through binding of growth factors to their cell surface receptors consisting of intracellular tyrosine kinase domains [2]. Human epidermal growth factor receptor 2 (HER2), known as Erythroblastosis homolog B2 (ErbB-2), is a transmembrane tyrosine kinase receptor and a member of the EGFR family. Overexpression of HER2 as one of the most important tumor associated antigens (TAA) is usually linked with increased tumor cell proliferation, tumor invasiveness, and angiogenesis [3]. Meanwhile, HER2 specific antitumor therapy has been well established as an efficient and highly selective strategy for treatment of some neoplasms like HER2-positive breast cancer [4]. Anti-HER2 trastuzumab and pertuzumab monoclonal antibodies (mAbs) are examples of FDA-approved adjuvants which are used in combinational therapy or monotherapy to target and kill tumor cells in early breast cancer [5, 6]. Moreover, anti-HER2 antibodies in the treatment of various cancers have shown great developments [7].

While potent, success in the cancer treatments based on these antibodies has been limited due to the low stability in vivo, off-target toxicity and raised drug resistance in patients with progressive tumors [8]. For example, resistance in tumor cells due to the low level of antibody-associated apoptosis can reduce the efficacy of treatment [9]. Additionally, the large size of antibodies greatly reduces their effectiveness, leading to their poor penetration into the tumor site or cells [10]. Therefore, higher doses of therapeutic antibodies are needed to compete with the serum IgG, which can cause severe side effects due to off-target bindings [11].

Overtime, delivering toxic drugs to tumor cells by targeting cancer specific cell-surface molecules is a key approach in cancer treatment that have minimal side effects on normal cells. For this purpose, antibody–drug conjugates and recombinant immunotoxins (rITs) are utilized. These compounds have two parts: a part that identifies the target molecule and the second part that has cytotoxic properties [12]. The rITs are obtained from the protein toxins of bacterial, plant, or human origin. Most of targeted therapies such as tyrosine kinase inhibitors (TKIs) inhibit tumor-supportive signaling pathways, however, acquired mutations can induce drug resistance. Unlike TKIs, rITs show less drug resistance because the applied toxins directly induce killing mechanism in the target tumor cells regardless of tumor mutations [13]. Herein, we review different methods of HER2-based cancer targeted therapy. Then recent HER2-based rITs studies and their potentials and drawbacks will be described in detail.

## HER2 signaling in human cancers

**Fig. 1:**
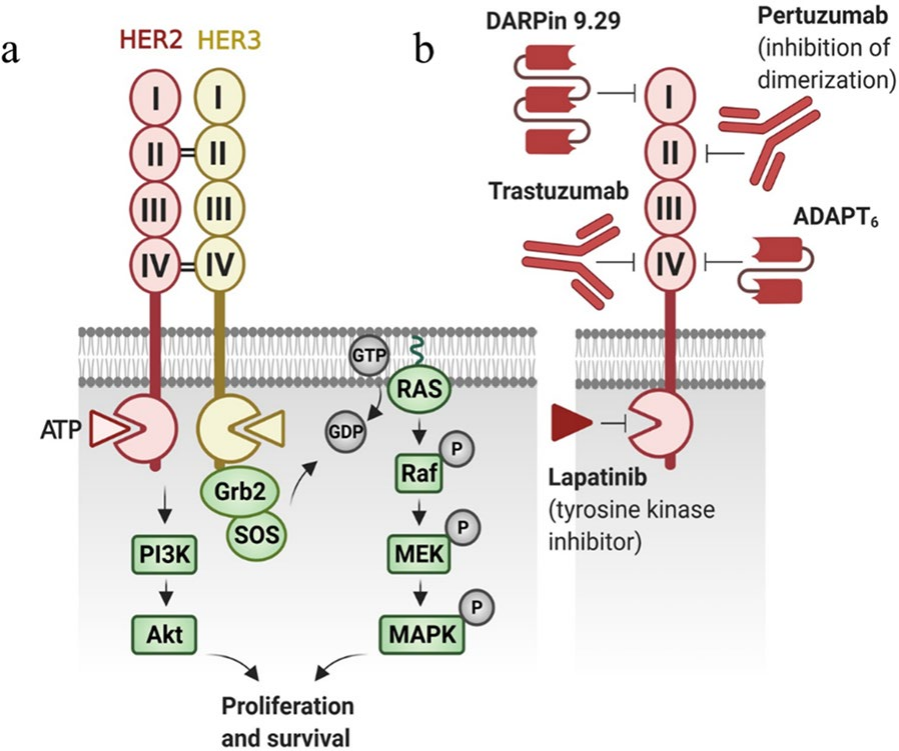
HER2 signaling and targeting agents. **a** HER2 antigen comprises an extracellular region (I–IV), a transmembrane region and an intracellular tyrosine kinase region. EGFR ligands interact to binding sites of the receptors which is followed by inducing receptor homo- or heterodimerization, C terminus phosphorylation and activation of the cytoplasmic tyrosine kinase for inducing downstream MAPK and PI3K/Akt signaling pathways. **b** According to HER2 structure and function, immuno therapeutic agents have been developed to target ECD I (e.g., DARPin), II (e.g., pertuzumab), IV (e.g., trastuzumab and ADAPT_6_), and tyrosine kinase region (e.g., lapatinib). *ECD* extracellular domain, *MAPK* mitogen-activated protein kinases, *PI3K* phosphatidylinositol-3-kinase

The members of human EGFR family (ErbB) are type-I transmembrane proteins that include HER1, HER2, HER3, and HER4 [14]. HER2 antigen is a protein with 1255 amino acids and comprises three regions including, an extracellular region, an amphipathic transmembrane region and an intracellular tyrosine kinase region. The N-terminal extracellular region of HER2 includes four domains, I, II, III and IV. Various ligands can potentially interact to the binding sites of extracellular domain I (ECD I) and ECD III of EGFR family receptors except HER2. ECD II and ECD IV are cysteine-rich regions and contribute in the homo- and heterodimerization [15]. The intracellular protein tyrosine kinase region comprises a C-terminal tail bearing tyrosine phosphorylation sites [16]. Briefly, ErbB family-ECD binds to EGF-related ligands followed by induction of receptor heterodimerization with HER2 that results in autophosphorylation of specific C-terminal tyrosine residues [17]. This autophosphorylation provides binding sites for proteins containing SH2 or PTB domains such as adaptor proteins (Shc, Crk, Grb2 and Grb7), kinases (Src, Chk and PI3K), and the protein tyrosine phosphatases (SHP1 and SHP2) [18]. Consequently, this processes induce several downstream cell proliferation and survival signaling pathways such as RAS/MAPK (rat sarcoma/mitogen-activated protein kinase), and phosphatidylinositol-3-kinase (PI3K)/Akt [19] (Fig. 1a).

HER2 is an orphan receptor for which a natural specific ligand has not been found yet [20]. It is assumed that its signaling occurs as a coupled receptor in heterodimerization with other ErbB family members [21]. Indeed, HER2 prefers to form heterodimerization with other ErbB members. The exposed ECD II of HER2 binds to other ligand-bound ErbB receptors with increased ligand affinity because of their lower dissociation rate [22]. The formed heterodimers induce prolonged activation of downstream signaling pathways [23]. Hence, the HER2/ErbB heterodimers boost downstream signaling more than other ErbB family homodimers [24].

Members of ErbB family are essential for proliferation and differentiation of fetal tissues as well as adult body tissues. However, their excessive expression causes the overactivation of above-mentioned downstream signaling pathways (e.g., MAPK and Akt/PI3K) leading to cell proliferation and differentiation, angiogenesis, and apoptosis blockade, which are linked with pathogenesis and progression of a large number of solid tumors [25]. Overexpression or amplification of HER2 oncogene have been shown in 25–30% of breast cancers, and 20–24% of gastric or gastro-esophageal junction cancers [26]. Up to 40–100-fold overexpression of HER2 protein and up to 25–50 copies of HER2 gene amplification have been shown in breast cancers [27]. Evidences suggest that overexpression or amplification of HER2 have been found in various cancers such as ovary, cervix, bladder, gallbladder and pancreas [28–32]. HER2-positive tumors are linked with an aggressive phenotype and poor clinical outcome [33]. Therefore, HER2 specific antitumor immunotherapies have been well established as described in the fallowing section.

## HER2-based targeted therapies

So far, several methods of HER2-based targeted therapy are developed (Table 1). Trastuzumab (Herceptin®) is a humanized mAb for HER2-positive breast cancer treatment [34]. Trastuzumab binds to ECD IV of HER2 (Fig. 1b), and prevents HER2–HER3 heterodimerization and tyrosine phosphorylation of HER2, and thus, suppresses PI3K/Akt activity resulting in inhibition of cancer cell proliferation [35, 36]. Trastuzumab also binds to the Fcγ III receptor on immune cells and activates antibody-dependent cellular cytotoxicity (ADCC) process [37]. Pertuzumab (Perjeta®) binds to the ECD II of HER2 (Fig. 1b), and blocks its heterodimerization with other ErbB members as well as downstream signaling pathways [38]. The efficacy of mAbs is improved by engineering bispecific antibodies having the ability of targeting HER2 positive tumor cells and immune cells concurrently (Table 1). For example, Ertumaxomab with trifunctional structure can simultaneously target HER2-expreesing cancer cells and CD3 antigens on T cells while its Fc region is identified by Fcγ type I/III receptor on tumor-suppressive immune cells [39]. Furthermore, the pharmacokinetic profile of utilized mAbs could be improved using the antibody-toxin conjugates, in which cytotoxic molecules kill tumor cells directly without induction of ADCC. Trastuzumab-based conjugate trastuzumab–emtansine (T-DM1, Kadcyla®), was approved in 2013 and has significantly improved the prognosis of breast cancer patients [40].

**Table 1:** HER2-based immunotherapy in clinic

Therapeutics	Features	Status	Tumor type	Refs.
Antibody				
Trastuzumab	ECD IV of HER2 binding humanized monoclonal antibody	FDA approved	HER2-positive breast cancer	[34]
Pertuzumab	ECD II of HER2 binding humanized monoclonal antibody	FDA approved	HER2-positive breast cancer	[38]
Margetuximab	Increased binding to activating Fcγ receptor IIIA (CD16A) and decreased binding to inhibitory Fcγ receptor IIB (CD32B)	FDA approved	HER2-positive BC, gastric cancers and gastro-esophageal junction cancer	[41]
BTRC4017A	T-cell dependent (CD3) bispecific antibody	Phase I (NCT03448042)	HER2-positive solid tumors	[42]
PRS-343	T-cell dependent (CD137) bispecific antibody	Phase I (NCT03330561, NCT03650348)	HER2-positive solid tumors	[43]
GBR-1302	T-cell dependent (CD3) bispecific antibody	Phase I (NCT02829372)	Breast cancer	[44]
ZW25	Bispecific antibody against two distinct HER2 epitopes	Phase I (NCT02892123) Phase II (NCT04513665)	Breast cancer, endometrial cancer, carcinosarcoma	[45]
Antibody–drug conjugate				
Trastuzumab– emtansine	Trastuzumab antibody linked to anti-mitotic agent, mertansine	FDA approved	HER2-positive metastatic breast cancer	[46]
ARX788	HER2 targeting mAb linked to the AS269 (a highly potent tubulin inhibitor)	Phase I (NCT02512237)	HER2-positive metastatic breast cancer	[47]
DS8201a	Trastuzumab–deruxtecan (DNA topoisomerase I inhibitor)	Phase I (NCT04042701)	Advanced/metastatic NSCLC	[48]
MEDI4276	Bispecific antibody targeting two different HER2 epitopes, conjugated with MMETA (a potent tubulysin-based microtubule inhibitor)	Phase I/II (NCT02576548)	HER2 positive breast cancer	[49]
MM302	Pegylated liposomal doxorubicin formulation, with anti-HER2 antibody fragments coupled to its surface.	Phase I (NCT01304797)	HER2-positive breast cancer	[50]
PF-06804103	Anti-HER2 monoclonal antibody conjugated with the cytotoxic agent Aur0101	Phase I (NCT03284723)	HER2 positive breast cancer and gastric cancer	[51]
SYD985	Trastuzumab–duocarmazine	Phase I (NCT02277717) Phase II (NCT04205630) Phase III (NCT03262935)	Metastatic breast cancer	[52]
XMT-1522	Anti HER2 IgG1 conjugated with the tubulin inhibitor AF-HPA	Phase I (NCT02952729)	Advanced breast cancer, gastric cancer and NSCLC	[53]
Tyrosine kinase inhibitor				
Lapatinib	HER1 and HER2 tyrosine kinases inhibitor	FDA approved	Metastatic breast cancer	[54]
Neratinib	HER1, HER2, and HER4 tyrosine kinases inhibitor	FDA approved	Early-stage HER2-positive breast cancer	[55]
Afatinib	ErbB family tyrosine kinases inhibitor	FDA approved	Metastatic NSCLC	[56]
Ibrutinib	ErbB family tyrosine kinases inhibitor	FDA approved	CLL, MCL, DLBCL, MM, FL and WM	[57]
Pyrotinib	HER1, HER2, and HER4 tyrosine kinases inhibitor	Phase I (NCT01937689)	Breast cancer	[58]

*CLL* chronic lymphocytic leukemia, *FL* follicular lymphoma, *MCL* mantle cell lymphoma, *DLBCL* diffuse large B cell lymphoma, *MM* multiple myeloma, *NSCLC* non-small cell lung cancer, *WM* waldenstrom’s macroglobulinemia

TKIs can compete with the ATP for binding to the ATP-binding domain of protein kinases. This competition prevents phosphorylation and further activation of the tyrosine kinase pathways, resulting in apoptosis and reduction of cellular proliferation [59]. In this context, Lapatinib (Fig. 1b), a reversible inhibitor for HER2 and EGFR, has been approved by FDA since 2007 for treatment of HER2-positive metastatic breast cancer [60] (Table 1).

Cancer vaccines trigger anti-tumor responses mediated by immune effectors such as cytotoxic T lymphocytes (CTLs) (CD8^+^T cells), CD4^+^T cells and antibodies against tumor cells. Peptides derived from different parts of HER2 molecule can be used as peptide vaccines. Several peptide vaccines have been developed for HER2 positive cancers such as AVX901 and E75 [61]. Initial clinical evaluations show minimum dose-limiting toxicity in the use of these vaccines, demonstrating their safety in HER2-positive breast cancers treatment [61].

Chimeric antigen receptors (CARs) are genetically modified receptors that contain an extracellular tumor-associated antigen (TAA) binding domain (e.g., single-chain variable fragment, scFv) with intracellular domains of co-stimulatory factors such as CD137 and/or CD28 and CD3ζ domain as activating domain [62]. Adoptive T cell transfer with CAR is an interesting cancer treatment strategy which has been evaluated to treat several HER2 positive glioblastoma and sarcoma [63, 64]. The use of HER2-CAR T-cell for the immunotherapy of HER2 positive sarcoma demonstrated the safety of this method, although no appropriate HER2-CAR T-cell expansion was observed [65]. Another study showed that chemotherapy-induced lymphodepletion provides expansion of HER2-CAR T cells in advanced sarcoma patients [66].

While potent, 25% of patients with early HER2-expressing breast cancer experience tumor recurrence after anti-HER2 mAbs therapy [40]. Furthermore, the acquisition of resistance in tumor cells is the main drawback of TKIs. Even in patients with the highest sensitivity to TKIs, tumor cells can gain resistance to these therapeutics by self-regulation [9]. The therapeutic efficacy of cancer vaccines is limited by the deficiency of T-cell activation and their suppression by immunosuppressive effectors in the tumor microenvironment. CAR T-cell therapy has been successful in hematological malignancies, nevertheless, the clinical outcomes in solid tumors have been controversial, which may be due to immunosuppressive tumor microenvironment and heterogeneous expression of TAAs [67]. These limitations and challenges conduit researchers to study and evaluates therapeutic potential of other agents such as immunotoxins as reviewed below.

## HER2-specific immunotoxins

Immunotoxins are recombinant proteins that contain a modified toxin along with a tumor specific ligand (Table 2). The cell surface receptors targeting moiety is usually a fragment of a mAb. rIT binds to tumor cell surface receptors through the tumor-specific ligand, then enters the cell by endocytosis [68]. The first attempts to target EGFR family was the use of recombinant fusion proteins made from the catalytic domains of *Pseudomonas* exotoxin A (PEA) or *Diphtheria* toxin in combination with natural EGFR ligands (TGF-α or EGF) [69, 70]. One approach for production of immunotoxins is chemical conjugation of selected toxic and targeting moieties. A major drawback of the chemical conjugation procedure is the heterogeneity of derived components, which complicates the production and purification processes. Loss of functional characteristics and difficulty in synthesis are other disadvantages of chemical conjugation [71]. Overcoming these limitations, rITs are produced based on genetic engineering or recombinant DNA technology by fusion of modified toxins and cell targeting fragments.

**Table 2:** HER2-based immunotoxins in cancer therapy

Immunotoxin	Targeting moiety	Toxic moiety	Origin of toxic moiety	Additional attribute	Mechanism of action	Refs.
***PEA-based rIT***						
4D5scFv-PE40	4D5scFv	PEA, PE40	*Pseudomonas aeruginosa*	–	Mono-ADP-ribosyltransferases	[71]
HER2-PE25-X7	Z_HER2:2891_	PEA, PE25-X7	*Pseudomonas aeruginosa*	–	Mono-ADP-ribosyltransferases	[72]
ADAPT_6_-ABD-PE38X8	ADAPT_6_	PEA, PE38X8	*Pseudomonas aeruginosa*	ABD	Mono-ADP-ribosyltransferases	[73]
Z_HER2:2891_-ABD-PE38X8	Z_HER2:2891_ Affibody	PEA, PE38X8	*Pseudomonas aeruginosa*	ABD	Mono-ADP-ribosyltransferases	[74]
Z_HER2:2891_-ADAPT_6_-ABD-PE25	Z_HER2:2891_ Affibody, ADAPT_6_	PEA, PE25	*Pseudomonas aeruginosa*	ABD, dual-targeting domain	Mono-ADP-ribosyltransferases	[75]
5F7-PE24 X7	5F7 sdAb	PEA, PE24 X7	*Pseudomonas aeruginosa*	G4S spacer	Mono-ADP-ribosyltransferases	[76]
11A4-PE24 X7	11A4 sdAb	PEA, PE24X7	*Pseudomonas aeruginosa*	G4S spacer	Mono-ADP-ribosyltransferases	[76]
47D5-PE24X7	47D5 sdAb	PEA, PE24X7	*Pseudomonas aeruginosa*	G4S spacer	Mono-ADP-ribosyltransferase	[76]
DARPin-LoPE	DARPin	LoPE	*Pseudomonas aeruginosa*	–	Mono-ADP-ribosyltransferase	[77]
***RIP-based rIT***						
RTA-4D5-KDEL		Ricin	*Ricinus communis*	ER-targeting peptide KDEL	*N*-Glycosidase	[78]
4D5/rGel	4D5scFv	Gelonin	*Gelonium multiflorum*	**–**	*N*-Glycosidase	[79]
Fab–Gelonin	Trastuzumab Fab	Gelonin	*Gelonium multiflorum*	Sortase A trasnpeptidase	*N*-Glycosidase	[80]
Trastuzumab–saporin	Trastuzumab	Saporin	*Saponaria officinalis*	–	*N*-Glycosidase	[81]
T-CUS245C	Trastuzumab	CUS_245C_	*Cucurbita moschata*	–	*N*-Glycosidase	[82]
***ImmunoRNase***						
ScFv 4D5-dibarnase	4D5scFv	Barnase	*Bacillus amyloliquefaciens*	**–**	RNase activity	[83]
hERB-hRNase	anti-ErbB-2 scFv	HP-RNase	Human	–	RNase activity	[84]
Erb-hcAb-RNase	Erb-hcAb	HP-RNase	Human	–	RNase activity, ADCC, CDC	[85]
ERB–HP-DDADD-RNase	Erbicin scFv	HP-RNase	Human	–	RNase activity	[86]
***Immunoapoptotin***						
GrbR201K-scFv1711	scFv	GrB	Human	–	Serine protease	[87]
GrB-4D5-26	4D5 scFv	GrB	Human	pH-sensitive peptide 26	Serine protease	[88]
GrB-Fc-4D5	4D5 scFv	GrB	Human	IgG Fc linker	Serine protease	[89]
GrB-FRP5	FRP5 scFv	GrB	Human	–	Serine protease	[90]
FRP5-ETA_252–366_-AIFΔ100	FRP5 scFv	AIF	Human	Translocation domain of PEA	Apoptosis effector	[91]
Immunocasp-6	e23sFv	Active caspase-6	Human	Translocation domain of PEA	Apoptosis effector	[92]
HER-PE-CP3	e23sFv	C-cp-3	Human	Translocation domain of PEA	Apoptosis effector	[93]
HusFv-Fdt-tBid	e23sFv	tBid	Human	Fdt linker	Apoptosis effector	[94]
e23sFv-TD-tBiD	e23sFv	tBid	Human	DT translocation domain	Apoptosis effector	[95]
***Immunophotosensitizer***						
4D5scFv-KillerRed	scFv	KillerRed	*Hydrozoa* jellyfish	–	ROS production	[96]
4D5scFv-miniSOG	scFv	miniSOG	*Arabidopsis*	–	ROS production	[97]
DARPin-miniSOG	DARPin	miniSOG	*Arabidopsis*	–	ROS production	[98]

*ABD* albumin binding domain, *ADCC* antibody-dependent cellular cytotoxicity, *AIF* apoptosis inducing factor, *CDC* complement-dependent cytotoxicity, *DT* diphtheria toxin, *Erb-hcAb* Erbicin-human-compact antibody, *GrB* granzyme B, *PEA* pseudomonas exotoxin A, *ROS* reactive oxygen species, *tBid* truncated Bid

Different types of toxins such as PEA, *Diphtheria* toxin (DT), ricin, gelonin, Granzyme B and RNases, have been studied as cytotoxic moiety used in rITs. Toxins used in rIT therapy usually have several domains: (i) cell-binding domain is responsible for attachment of toxins to the cell, (ii) translocation domain which transports the toxin into the cytosol of the target cell, and, (iii) catalytic domain that plays the role of cytotoxicity and cell death induction. To construct rITs, the cell-binding domain of toxins is removed, and then modified toxins are genetically fused to the variable fragment of an anti-TAA antibodies [99]. These anti-HER2 humanized antibodies derived from murine antibodies such as 4D5 [100], FRP5 [101], and E23 [102] are used as the targeting moiety in HER2-based therapeutics.

Better than whole sized mAbs, scFv is the best choice for the production of fusion proteins in targeted therapy. These molecules have the advantages of high permeating capability and feasibility of producing recombinant constructs. The scFv includes VH and VL sequences that are genetically linked to toxins by a linker. ScFv molecules lack the Fc domain, so they do not interact with Fc receptors in normal cells [103]. For example, trastuzumab-derived 4D5scFv is widely used in the development of HER2-specific rITs. 4D5scFv has high thermodynamic stability in serum and binds to HER2 with high affinity [100].

In addition to antibodies, non-immunoglobulin scaffold proteins like Designed Ankyrin Repeat Proteins (DARPins) can be used in this targeted therapy [104]. DARPins are small single-chain scaffold proteins that can be designed using protein engineering techniques for binding to specific targets [105]. Similar to scFv, the small size of DARPins improves their cellular penetration compared to the antibodies. In addition, the lack of free cysteine in DARPins structure facilitate their production in prokaryotic organisms in efficient manner. They are also less immunogenic and do not appear to stimulate T cell-independent immune responses [106]. As an example, DARPin_9-29 that binds to the ECD I of HER2 is currently being studied in HER2-based targeted therapy [107] (Fig. 1b). Since DARPin_9-29 does not compete with 4D5 or pertuzumab recognizing epitopes [107], these molecules can be combined for HER2 targeting.

Affibodies are another non-immunoglobulin scaffold proteins that can specifically interact with several tumor antigens such as HER2, IGF-1R and EGFR [108–110]. The ease of engineering, molecular size below 10 kDa, and short plasma half-lives of affibodies are favorable properties that extended their utilization in diagnostic imaging of HER2-expressing tumors following labeling with near infrared fluorescent probes and radiometals [111]. However, the small size of Affibodies (58 amino acids, 7 kDa) leads to quick glomerular filtration and renal accumulation of therapeutics. This issue can be addressed by genetically fusion of HER2-specific affibody molecule to the albumin-binding domain (ABD) that results in extension of affibody half-life and induction of antitumor effect in a micrometastatic model of breast cancer [112]. For example, Z_HER2:2891_, a HER2 specific affibody with high binding affinity, has been fused to ABD resulting in enhanced serum half-life of the affibody and also higher in vivo efficacy [74].

ABD-derived affinity proteins (ADAPTs) are another group affinity proteins consisting of 46 amino acids (5 kDa). ADAPT_6_ is the most widely studied variant in HER2 targeted therapy which binds to the ECD IV of HER2 with a *K*_*D*_ of 0.5 nM [113] (Fig. 1b). The small size of ADAPTs and affibodies leads to a more efficient accumulation of therapeutics in solid tumors. Similar to affibodies, the small size of ADAPTs reduces their half-life in blood circulation, a feature that potentiates them for tumor imaging [114, 115].

Nonetheless, for therapeutic application, short half-life and fast blood clearance of fusion proteins require more injections. The rITs comprising ADAPTs combined with toxins, are small enough to be cleared from the circulation quickly. Adding an ABD to these target molecules associates them with albumin and increases their half-life in the bloodstream [113]. Recently, bioinformatics approaches have been considered to reduce the undesirable properties of toxin and targeting molecules of immunotoxins such as immunogenicity and increase their efficiency [116].

### *Pseudomonas* exotoxin A-derived immunotoxins

**Fig. 2:**
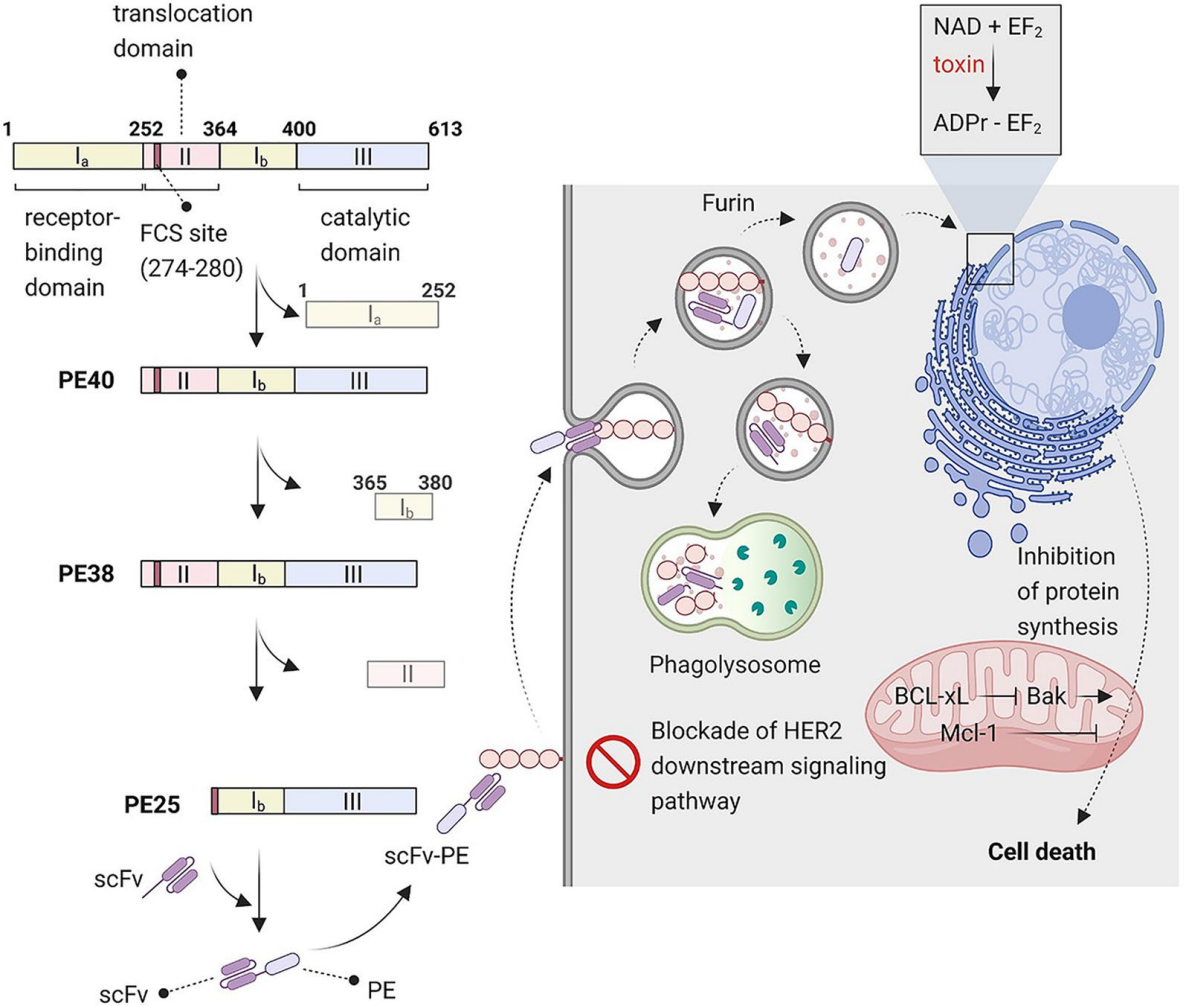
PEA-derived rITs for HER2 targeted therapy. The Ia domain (aa 1–252) in the N-terminus of PEA polypeptide is responsible for binding to the target cell, and domain II (aa 253–364) transports toxins from the cytoplasmic membrane into the cell. The domain III (aa 405–613) and last 4 residues of domain Ib are responsible for inhibition of protein synthesis. Domain Ia is removed to produce a PE40 (40 kDa). PE38 (38 kDa) is produced through removing domain Ia and amino acids 365–380 of domain Ib. PE25 (25 kDa) is produced through removing domain II from PE38, except FCS. For cancer therapy, PEAs are fused with a HER2-targeting scFv to produce rIT s. scFv mediates binding of rIT to HER2. After endocytosis, the toxin moiety released by furin cleavage at FSC, and through ADP-ribosyltransferase activity suppress the protein synthesis to induce cancer cells death. *PEA* *Pseudomonas* exotoxin A, *rIT* recombinant immunotoxin, *FCS* furin cleavage site

*Pseudomonas* exotoxin (PE) A is a highly toxic mono-ADP-ribosyltransferase that transfers ADP ribose from NAD to elongation factor 2 (EF2) causing EF2 inactivation, and thus irreversibly inhibits protein translation [117]. PE polypeptide chains have several distinct functional and structural domains. The Ia domain in the N-terminus of polypeptide chain containing amino acids 1–252, is responsible for identifying CD91 (alpha2-macroglobulin receptor) and binding to the target cell. Domain II contains amino acids 253–364 and transports toxins from the cytoplasmic membrane into the cell. Domain Ib contains amino acids 365–404 and has no specific role in toxin function. The last 4 residues of domain Ib along with domain III (including amino acids 405–613) are responsible for cytotoxicity and inhibition of protein synthesis and cell death induction by ADP-ribosyltransferase activity [118]. Domain Ia is not essential in the development of the rIT and is removed and replaced by a specific TAA ligand. Deletion of domain Ia yields a 40 kDa protein called PE40. By removing domain Ia and amino acids 365–380 of domain Ib, a 38Kd protein called PE38 is obtained that has no impact on toxin function [119] (Fig. 2).

C-terminus of the polypeptide chain has the REDLK motif (aa 609–613). The terminal lysine (aa 613) of this motif can be removed in the extracellular environment, leading to REDL motif (aa 609–612), which allows the exotoxin to bind to the golgi apparatus KDEL receptor through intracellular trafficking. To increase PE cytotoxic activity and improve its intracellular trafficking, REDLK can be replaced with KDEL [120].

PE-based rITs are internalized into endosomes after binding to HER2 antigen. These rITs undergo proteolytic processing during intracellular transport. The transport of the toxin fragment derived from PE-based rITs is conducted similar to that of wild-type PE. Once entered to the endosomes, the rITs are cleaved by the furin proteases at the furin cleavage site to detach antibody fragment from the toxin. The toxin fragment is subsequently transferred to the Golgi apparatus where the REDL motif at the C-terminus binds to the KDEL receptor and transports it reterogradely into the endoplasmic reticulum. The toxin then enters the cytosol from the endoplasmic reticulum and inhibits protein synthesis [121] (Fig. 2).

4D5scFv-PE40 is a HER2 specific rIT that contains the 4D5scFv fragment and PE40 of PEA as the effector module [122]. A flexible 16 residue linker from hinge region of mouse IgG3 connects 4D5scFv to PE40. The specific toxicity of 4D5scFv-PE40 against HER2-positive cells has a very low IC50 value in the picomolar range, whereas in HER2-negative cells this value is much higher [123].

Guo et al. [72] developed a new rIT, called HER2-PE25-X7 through removing most of the domain II, except Furin protease cleavage site, and introducing seven-point mutations in domain III of PE38. These modifications greatly reduced the immunogenicity and off-target toxicity of the HER2-PE25-X7 while maintaining the therapeutic potency of original PE38 [72].

Recently, several rITs based on deimmunized PE38 and PE25 variants have been developed that their targeting elements consist of affibodies and ADAPTs [75]. For example, Liu et al. [74] produced and characterized an rIT consisting of a deimmunized PE38 version. PE38X8 was genetically fused to Z_HER2:2891_ (HER2 specific affibody molecule) and an ABD for half-life extension. In another attempt, This group. designed and evaluated some ADAPT-based immunotoxins, including ADAPT_6_-ABD-PE38X8 and ADAPT_6_-ABD-PE25. They found that Z_HER2:2891_-ABD-PE38X8 (K_D_ = 5 nM) exhibited a higher binding affinity to HER2 compared to the ADAPT_6_-ABD-PE38X8 (K_D_ = 26 nM) [113].

### Ribosome inactivating protein-based immunotoxins

Two types of plant toxins have been exploited in immunotoxin-based therapeutics: (i) holotoxins (e.g., ricin, mistletoe lectin, and abrin), which are known as ribosome inactivating proteins (RIPs) class II with catalytic and binding domains, and (ii) hemitoxins (e.g., gelonin, PAP, bouganin, and saporin), that are referred to as ribosome inactivating proteins class I with only catalytic domains [124] (Table 2).

**Fig. 3:**
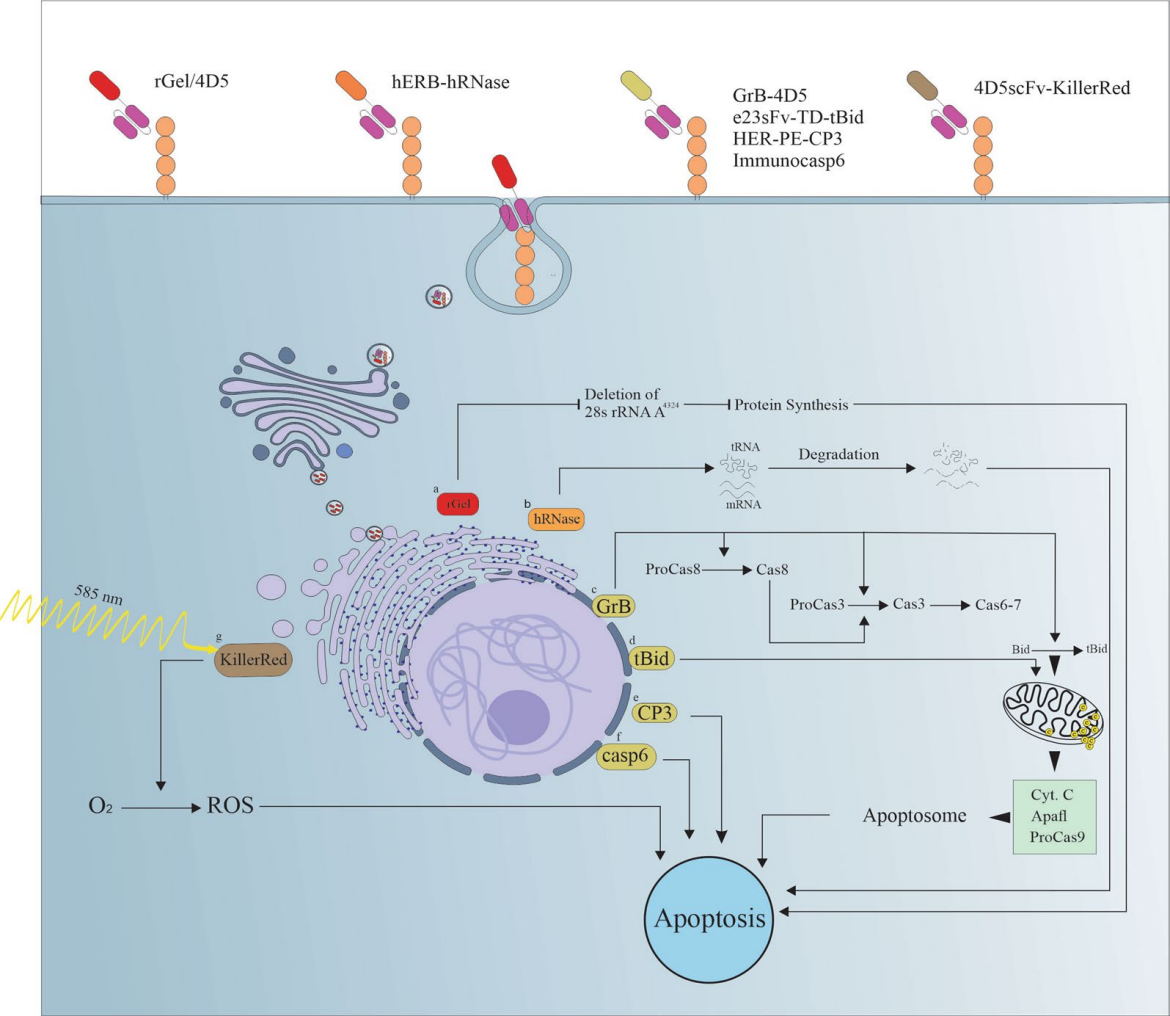
Different mechanisms in which the immunotoxins induce apoptosis in cancer cells. **a** rGel as a RIP inhibits the protein synthesis by removing A^4324^ of eukaryotic 28 S ribosomal RNA. **b** hERB-hRNase induces the tRNA and mRNA degradation which followed by suppression of protein synthesis and induction of apoptosis. **c**–**f** GrB-4D5, e23sFv-TD-tBid, HER-PE-CP3, and immunocap-6 are immunoapoptotins which their effector moieties (i.e., GrB, tBid, CP3 and cap-6) mediate apoptosis in cancer cells directly. **g** KillerRed as a photosensitizer promotes the production of ROS under laser irradiation to induce apoptosis. *CP3* caspase3, *Cyt. C* cytochrome C, *GrB* granzyme B, *RIP* ribosome inactivating proteins, *ROS* reactive oxygen species, *tBid* truncated Bid, *TD* diphtheria toxin linker

*Gelonium multiflorum* seeds-extracted gelonin is a type I (RIP) which has *N*-glycosidase property and inactivates the ribosome by removing A^4324^ from eukaryotic 28 S ribosomal RNA [125]. Members of the type I RIP family have only catalytic polypeptide chains, while type II RIPs have two chains, the A-chain as the catalytic domain and the B-chain as the cell-binding domain [126]. Recombinant gelonin (rGel) is a 29-kDa single-chain protein with strong cytotoxic properties that has been considered for the production of conjugates in the treatment of cancers [127, 128]. In various studies, anti-Her2-rGel-based fusion proteins have been designed using flexible linkers between an anti-Her2 molecule and rGel. For instance, rGel toxin was fused to the human scFv C6.5 and murine scFv e23 with a flexible G4S linker and furin cleavage sites containing the linkers Fpe (TRHRQPRGWEQL) and Fdt (AGNRVRRSVG). C6.5-rGel constructs containing G4S linker possess higher in vivo stability, and thus, presented more efficiency in tumor growth inhibition than those containing furin linkers [129]. In another study, affinity mutation of anti-HER2 scFv C6.5 (ML3-9, MH3-B1, and B1D3) in rGel-based rITs increased the affinity, internalization capability, and autophagic cytotoxicity [130]. Comparison of bivalent chemical Herceptin/rGel conjugate and monovalent rITs in two directions including 4D5/rGel (rGel is fused to 4D5 by VH) and rGel/4D5 (rGel is fused to 4D5 by VL) showed that they all have the same affinity for Her2-expressing cancer cells, although their antitumor activity is different [79]. Herceptin/rGel conjugate and rGel/4D5 orientation construct had higher antitumor efficacy than 4D5/rGel, which could be attributed to their better intracellular absorption [79] (Fig. 3a).

*Saponaria officinalis-*derived Saporin is a RIP class I that has been considered for its appropriate properties such as strong cytotoxic effects, high thermal stability and denaturation resistance [131]. Cucurmosin (CUS) is another type I RIP cytotoxin isolated from *Cucurbita moschata* (pumpkin). Conjugation of trastuzumab to modified CUS (CUS_245C_), T-CUS245C, showed a potent cytotoxicity on HER2-positive SK-OV-3 cells with a IC_50_ of 10 pM [82]. *Luffa cylindrica* seeds*-*derived Alpha luffin protein is another type I RIP, which recently showed RNase and DNA glycosylase activity when fused to an anti-HER2 scFv [132].

The castor bean, *Ricinus communis-*isolated Ricin is a type II RIP containing two polypeptide chains, RTA and RTB, which are linked by a disulfide bond. RTA is a 263 amino acid polypeptide that removes G^4323^ at 28 S rRNA by *N*-glycosidase activity leading to inhibition of protein synthesis and cell death induction [133]. RTB is a lectin that can bind to the cell surface via galactose residues of membrane glycoproteins and glycolipids fallowed by entering ricin into the cells through endocytosis process [134, 135]. Anti-p185^HER-2^‐RTA induced apoptosis in HER‐2 positive gastric cancer cells (SGC7901‐HER‐2+) by raising caspase‐3 and caspase‐9 activity [136].The toxicity of ricin is only exploited when transported to the cytoplasm. Addition of the KDEL sequence to ricin increases its transportation to the cytoplasm [137]. In this regard, fusion of KDEL to the C-terminal of RTA-4D5 increased its anti-cancer effect on SKOV-3 ovarian cancer cells by 440- and 28-fold compared to the RTA and RTA-4D5, respectively [78].

### Anti-HER2 immunoRNases

RNases are heterogeneous group of RNA hydrolyzing enzymes that can have cytotoxic properties. Several types of RNases such as Barnase, Binase, Ranpirnase, human pancreatic RNase 1 (HP-RNase 1), bovine pancreatic RNase A, bovine seminal RNase, human eosinophil-derived RNase and angiogenin have exhibited potent toxicity on tumor cells [138–141] (Table 2). *Bacillus* a*myloliquefaciens-*derived Barnase and *Bacillus intermedius*-derived Binase are two structurally similar RNases with comparable catalytic activity, which are widely used in the development of rIT-based therapies [142]. The scFv 4D5-dibarnase is a Barnase-based immunoRNase consisting of two Barnase molecules that are fused to the scFv of 4D5. The scFv 4D5-dibarnase selectively binds to the HER2-positive cells and is internalized through receptor-mediated endocytosis [143]. This immunoRNase has showed a specific cytotoxicity with IC50 in the nanomolar range on HER2 positive breast cancer cells [143]. Balandin et al. [83] showed that application of ten doses of 0.7 mg/kg scFv 4D5-dibarnase resulted in a 76% reduction of tumor growth in the xenograft models bearing SKBR-3 human breast cancer cells, without severe side effects.

Currently, human RNases are more popular due to their low immunogenicity. Human RNases are inherently limited in cytotoxicity, but their cytotoxicity can be enhanced when combined with a cancer-specific ligand. One of the first human immunoRNases, called hERB-hRNase, was constructed by combining anti-HER2 scFv and HP-RNase. This immunoRNase presented a high specificity and affinity to HER2-positive tumor cells and remarkably prevented tumor growth in mice models [84] (Fig. 3b). Erb-hcAb-RNase is another rIT which is a fusion of HP-RNase to anti-HER2 compact antibody. Compact antibody contains two scFv molecules (Erb-hcAb) linked to the human IgG1 Fc region. Therefore, Erb-hcAb-RNase presented ADCC and complement-dependent cytotoxicity (CDC) activities, as well as RNase-based cytotoxicity against HER2 overexpressing tumor cells [85]. While potent, the physiological conditions of the cell and the maintenance of balance in the ribonucleic acid content exploit endogenous obstacles to the RNases activity and thereby reduce their therapeutic efficacy [144]. The natural interaction of RNases with the endogenous inhibitors can be prevented by introducing new mutations [145]. ERB-HP-DDADD-RNase is one of these cases which was developed by the fusion of a resistant variant of HP-RNase (HP-DDADD-RNase) with the anti-HER2 scFv Erbicin. In this approach, five residues that are crucial for binding of HP-RNase to the RNase inhibitors were mutated that endowed an inhibitor-resistance phenotype to the RNase, and improved its anti-proliferative activity [86].

### Anti-HER2 immunoapoptotins

Another type of complete humanization of rITs is named human cytolytic fusion proteins (hCFPs), originating from the cytotoxic endogenous human apoptotic-relevant enzymes fused to humanized antibody fragments. So far, various endogenous pro-apoptotic molecules such as granzyme B (GrB) [88], truncated Bid (tBid) [94], caspase-3 [93], caspase-6 [92, 146] and apoptosis-inducing factor (AIF) [147] have been utilized to induce apoptosis in tumor cells (Table 2).

Granzymes are pro-apoptotic serine proteases that expressed and stored in granules of CTLs and natural killer (NK) cells [148, 149]. Based on the time of discovery, five types of the granzymes have been identified so far and named as GrA, GrB, GrH, GrK, and GrM [150]. GrB is initially produced as a pro-apoptotic enzyme and activated by the removal of the N-terminal dipeptide Gly-Glu. Perforin acts as cell membrane-destroying protein by which the GrB released from CTLs and NK cells can enter to the cytosol and induce apoptosis in both transformed and virus-infected cells [151]. The efficacy of pro-apoptotic enzymes-based targeted therapy is limited by apoptosis-escape mechanisms. However, GrB induces apoptosis through various mechanisms to overwhelm these resistance mechanisms in tumor cells [152]. Therefore, GrB can be considered as a potential therapeutic approach to eliminate apoptosis-resistant tumor cells. For example, GrB fused to humanized anti-HER2 scFv, GrB-4D5, is an rIT against HER2 that showed specific tumor cell killing function in BT474 M1 cells [153] (Fig. 3c).

Normally, the perforin-produced pores in cell membrane mediate the internalization of GrB into cytosol of tumor cells. To improve the delivery efficacy, GrB has been linked to anti-HER2 scFv molecules. However, targeted antibodies can induce receptor-mediated endocytosis, which subsequently results in the GrB degradation in the lysosomes. Addressing this limitation, Cao et al. [88] developed a construct by fusing an anti-HER2 scFv to GrB with pH-sensitive peptide 26, that its converting configuration under acidic lysosomal condition induces lysosomal disruption as well as delivery of therapeutic construct into cytosol. GrB-Fc-4D5 is another immunoapoptotin which contains 4D5scFv fused to GrB using Fc fragment of IgG as a linker. This linker and dimerization capability eliminated the need for immunoapoptotin release from lysosomes and increased the half-life of this rIT in the bloodstream. Compared to GrB/4D5, GrB-Fc-4D5 dramatically activated the caspase-9, and inhibited the Akt phosphorylation that consequently induced apoptosis in cancer cells [89]. Due to the necessity of regulation of granzymes expression in human immunosurveillance system, they are strongly controlled by granzyme-regulating serpin, PI-9, which can affect GrB-based therapies. To overcome this obstacle, several PI-9 insensitive GrB mutants such as R28A, R28E, R28K, R201A, R201E, R201K have been engineered [154]. GbR201K-scFv1711 is an hCFP containing a PI-9-resistant mutant of GrB (R201K) and the human EGFR-specific antibody fragment scFv171. This insensitivity increased the therapeutic efficiency of the GbR201K-scFv1711 fusion protein in epidermoid cancer and rhabdomyosarcoma cells compared to those with the wild-type GrB [87].

Bid is a pro-apoptotic protein that requires cleavage by proteases such as caspase-8 to be activated. Truncated Bid (tBid) potently activates Bax or Bak, leading to mitochondrial dysfunction and release of other pro-apoptotic agents [155]. The tBid-based immunoapoptotin e23sFv-TD-tBid comprises the anti-HER2 e23sFv and tBid (15-kDa), which are linked by a 10-amino acid diphtheria toxin translocation domain as cleavable spacer (Fig. 3d). After entering to cell cytosol through endocytosis, this immunotoxin is translocated into the trans-Golgi network and exerts its cytotoxic activity with an irreversible effect on the mitochondria of target cells [95]. In another study, husFv03-Fdt-tBid and husFv04-Fdt-tBid immunoapoptotins were designed using humanized e23sFv, which demonstrated HER2 targeting and cytotoxic effects on HER2-positve tumor cells. Humanization of e23sFv (husFvs) increased its affinity to recombinant HER2 up to 94-fold. In addition, the immunogenicity of e23sFv was considerably reduced after humanization [94].

Caspases (cysteine-aspartic proteases) are pivotal enzymes involved in apoptosis pathways of mammalian cells. Apoptosis can be activated through extrinsic or intrinsic pathways. In the extrinsic pathway, stimuli such as TNF (tumor necrosis factor) or FASL (Fas ligand) bind to death receptors and activate caspase-8. In the intrinsic pathway, intracellular stimuli such as BIM or puma cause mitochondrial depolymerization and cytochrome C leakage into the cytosol, leading to the activation of caspase-9. Both pathways eventually activate effector caspases (i.e., caspase 3, 6, and 7), which cause cell death by affecting different proteins [156]. Caspase-3 comprises a prodomain at the N-terminus, a large subunit, and a small subunit at the C-terminus. By the activation of early caspases, caspase-3 is cleaved between these domains, leading to the release of the prodomain. The large and small subunits rearranged in an opposite direction, which small subunit is located at the N-terminus. This heterodimer is the active form of caspase-3 which can proceeds apoptosis. A synthetic caspase-3 form, C-cp-3, has been constructed, which possess similar enzymatic activity compared to the natural active caspase-3. HER-PE-CP3 is a caspase-3 based immunoapoptotin, consists of a secretion signal, e23sFv, the translocation domain (domain II) of PEA, and the constitutively active C-cp-3. This immunoapoptotin induced a selective apoptosis in HER2 overexpressing gastric cancer cells and inhibited the growth of human gastric tumor in xenograft models [93] (Fig. 3e). Immunocasp-6 is an immunoapoptotin designed by fusion of the anti-HER2 e23sFv, the domain II of PEA, and an active caspase-6. Injection of liposome-encapsulated pCMV-immunocap-6 into human osteosarcoma bearing BALB/c athymic mice showed that immunocasp-6 induced apoptosis in HER2-overexpressing osteosarcoma cells and remarkably prevented tumor growth and metastasis [92] (Fig. 3f).

### Immunophotosensitizers

Photodynamic therapy (PDT) is a therapeutic approach in which tumor cells are killed by the production of reactive oxygen species (ROS) using light-excited photosensitizers. ROS compounds like singlet oxygen, OH· and O_2_· radicals, with their extremely reactive and toxic properties can oxidize cellular components and hence cause cell destruction and death [157]. Photosensitizers can act through type I or type II photodynamic reactions. In type I reactions, in an initial step, a photo-induced electron transfers from a donor molecule to photosensitizer; then photosensitizer reduces molecular oxygen and generates superoxide anion. In type II reactions, energy transfers from excited photosensitizer to molecular oxygen directly, leading to the production of singlet oxygen [158].

Immunophotosensitizer (immunoPS)-based PDT is highly specific for two reasons. First, antibody fragments against specific receptors on tumor cells cause them to accumulate in the tumor region. Second, light is radiated only on the tumor zone, thus limiting ROS production in that area [159]. Due to the expression of HER2 in normal tissues like hepatocytes and vascular endothelium, the use of traditional HER2 immunotoxins are usually associated with side effects such as hepatotoxicity and vascular leak syndrome [160]. In immunoPSs, the cytotoxic effect is limited to the light radiation region. This is an advantage that can minimize such subsequent side effects [161].

Various protein photosensitizers have been developed and used in targeted cancer therapeutics such as KillerRed [162], KillerOrange [163] and miniSOG [164] (Table 2). KillerRed is a red fluorescent protein obtained by direct evolution of the non-fluorescent protein anm2CP from *Hydrozoa* jellyfish. Maximum excitation and emission of KillerRed are 585 and 610 nm, respectively [162]. 4D5scFv-KillerRed is a recombinant immunoPS that has been shown to be highly specific for HER2-overexpressing cells [165] (Fig. 3g). mini-Singlet Oxygen Generator (MiniSOG) is a green fluorescent flavoprotein and another type of photosensitizer that has been investigated in HER2-based therapeutic developments. This flavoprotein is derived from *Arabidopsis* phototropin 2, and produces singlet oxygen by blue light irradiation [164]. 4D5scFv-miniSOG, a HER2 targeting immunoPS, has more phototoxic effect than 4D5scFv-KillerRed. Depth of light penetration in target tissue is an important factor in the determination of photodynamic therapy’s efficiency. Accordingly, the limited penetration of blue light through the tissues narrowed the application of 4D5scFv-miniSOG in clinic, which can be improved using the fiber-optic light delivery systems [97]. DARPin-miniSOG is another immunoPS which has been obtained by fusing miniSOG and DARPin-9-29. Compared to the 4D5scFv-miniSOG, DARPin-miniSOG has a smaller size that enhances the receptor-mediated internalization and accumulation in the early endosomes and lysosomes, and thus induces less cytotoxicity [98].

## Recombinant immunotoxins in clinical trial

Denileukin diftitox (Ontak®) is the first FDA-approved immunotoxin containing IL-2 and a truncated form of diphtheria toxin (DAB389) that is used for the treatment of recurrent cutaneous T-cell lymphoma (CTCL) [166]. Another FDA-approved PE38-based IT is anti-CD22 moxetumomab pasudotox which is used in patients with relapsed/refractory hairy cell leukemia [167]. In solid tumor trials, Kreitman et al. [168], conducted a phase I trial (NCT00006981) to evaluate SS1P (an anti-mesothelin PE-based rIT) given by continuous infusion in chemoresistant mesothelin-expressing mesothelioma, ovarian, or pancreatic cancers. Immunogenicity was observed in 75% of patients, and 21% received a second cycle of treatment. As a single agent by continuous infusion, SS1P was well tolerated up to 25 µg/kg/d × 10 and showed evidence of moderate clinical function [168]. Oportuzumab monatox is a rIT containing anti-epithelial cell adhesion molecule (EpCAM) humanized scFv fused to a PE (ETA252–608). Kowalski et al. [169], performed a phase II study to assess the efficacy and tolerability of this rIT in patients with Bacillus Calmette-Guérin (BCG) refractory urothelial carcinoma in two distinct cohorts. A complete response to Oportuzumab monatox was seen in 41% (cohort 1) and 39% of patients (cohort 2) at 3-months of evaluation. Patients achieved a complete response had a recurrence time median of 274 and 408 days in cohorts 1 and 2, respectively. The results of this study showed that Oportuzumab monatox was effective and well tolerated in patients with BCG refractory urothelial carcinoma [169].

A phase I clinical trial conducted by Pai-Scherf et al. [170] showed that a HER2-specific erb-38 rIT causes hepatotoxicity in all patients with breast cancer. This side effect is explained by previous studies that have demonstrated the expression of HER2 at very low levels on the surface of hepatocytes, which can lead to off-target toxicity [160, 171]. ScFv (FRP5)-ETA contains scFv of anti-HER2 FRP5 antibody linked to truncated Pseudomonas exotoxin A (ETA). Minckwitz et al. [101], conducted phase 1 clinical study to determine the maximum tolerated dose and the dose-limiting toxicity of scFv(FRP5)-ETA in 18 patients with HER2 positive solid tumors. They did not observe hematologic, renal and cardiovascular toxicities, and concluded that the use of scFv (FRP5)-ETA up to a maximum tolerated dose of 12.5 µg/kg can be safe. In another study, MT-5111, an rIT containing Shiga-like Toxin A subunit, conjugated to an antibody-like targeting domain. This rIT could bind to HER2 and show strong cell cytotoxicity by permanently inactivation of ribosomes. Currently, MT-5111 is in the phase I clinical trial to determine its maximum tolerated dose (MTD), pharmacokinetics, efficacy, and immunogenicity in HER2-positive breast cancer patients (NCT04029922). So far, results have shown that MT-5111 is well tolerated and has no clinically significant cardiotoxicity [172]. However, the raised challenges such as ineffective penetration of rITs into tumor tissues and their neutralization by patients’ immune system has led to the application of only few rITs in clinical trials of solid tumors.

## Challenges and future prospects

Since the first demonstration of the HER2 targeted therapy concept in 1986, anti-HER2 mAbs are widely used and have shown clinical success meanwhile [5, 6]. However, there have been some doubts as to the role of HER2 as a single receptor in transducing cytoplasmic responses. Moreover, it has been found that a significant percentage of patients has no homogeneity between HER2 gene and protein [33, 173]. To address this, scientists have recently used a novel gene protein assay (GPA), which can simultaneously access HER2 gene copy number and protein levels on a single slide using bright-field microscopy [33, 173].

In the immunotoxins, the fragments of specific antibodies or ligands of overexpressed receptors on tumor cells deliver cytotoxic molecules to the target tumor cells [68]. Unlike solid tumors, in hematological malignancies the target cells are readily available for rITs and thus, the direct injection of these drugs into the bloodstream are more successful in treatment of hematologic cancers. The success of rITs against solid tumors depends on their successful delivery to the tumor site at optimal concentration [174]. The scFv molecules have a size of 25–30 kDa, and together with the toxic moiety, form an immunotoxin with suitable size for penetration into solid tumors’ environment [103]. However, clearance of immunotoxins containing a scFv from the bloodstream occurs rapidly, and their affinity for target molecules is relatively low due to their monovalency, which results in the low uptake of these molecules into the tumor mass [175]. To overcome these limitations, bivalent scFvs, bispecific scFvs, diabidies and minibodies have been used in the immunotoxin platforms [176].

The immunogenicity of the toxic and targeting moieties is another challenge for application of the majority of immunotoxins in preclinical and clinical settings [177]. Immunogenicity of the targeting moiety can be overcome by using humanized or fully human antibody fragments such as scFvs [178]. Furthermore, once administrated, the toxin part of rIT can induce the production of neutralizing anti-toxic antibodies in patients, which can be partially prevented by deleting or altering the B-cell or T-cell epitopes in the toxic moiety [68, [113, 179], or using immunosuppressive drugs along with immunotoxins [180]. As PE38 obtained from bacteria, it has high immunogenicity in patients with normal immune system. Elimination of potential B and T cell-recognizing epitopes in PE38 has led to the generation of various deimmunized variants such as PE38X8 [181]. Better than targeting component, the effector component of human origin such as proteases, kinases and RNases can be even more effective in reduction of rITs’ immunogenicity [84, 182, 183].

Once PE-based immunotoxins enter cancer cells, their efficacy is limited by multiple resistance mechanisms such as lysosomal destruction, impaired cleavage, and low level of pro-apoptotic proteins or elevation of anti-apoptotic proteins. These drawbacks could be addressed by optimizing selected antigens, and using immunotoxins combined with other agents that modulate lysosomal activity or induce the extrinsic apoptotic pathway [184]. Finally, one of the main limitations of immunoPSs such as KillerRed is their lower phototoxicity than chemical photosensitizers, which requires high doses of both irradiation and photosensitizers to achieve the desired cytotoxicity. This limitation compels researchers to construct novel KillerRed variants with sufficient phototoxicity. Even though, the excessive ROS-induced phototoxicity can also cause problems such as necrosis of healthy tissues around the tumor [185].

## Conclusion

Immunotoxins exhibit selectivity and potency for cancer therapies and may be clinically effective as a single therapeutic for targeting of cancer cells expressing a high level of HER2. The therapeutic effect of immunotoxins may be beneficial as part of a combined treatment with other agents that increase toxin killing activity and reduce immunogenicity. Of note, for selectin of combined agents, their possible antagonistic/side effects must be carefully considered. Future work will be required for evaluation of novel approaches like identification and elimination of mechanisms of resistance to HER2-based immunotoxin, in order to tailor immunotoxin therapy and improve the treatment response in clinical setting.

## Data Availability

Data sharing not applicable to this article as no datasets were generated or analyzed during the current study.
